# Genetic and Non-Genetic Inheritance of Natural Antibodies Binding Keyhole Limpet Hemocyanin in a Purebred Layer Chicken Line

**DOI:** 10.1371/journal.pone.0131088

**Published:** 2015-06-26

**Authors:** T. V. L. Berghof, S. A. S. van der Klein, J. A. J. Arts, H. K. Parmentier, J. J. van der Poel, H. Bovenhuis

**Affiliations:** 1 Animal Breeding and Genomics Centre, Department of Animal Sciences, Wageningen University, Wageningen, the Netherlands; 2 Adaptation Physiology Group, Department of Animal Sciences, Wageningen University, Wageningen, the Netherlands; National Cancer Institute, UNITED STATES

## Abstract

Natural antibodies (NAb) are defined as antibodies present in individuals without known antigenic challenge. Levels of NAb binding keyhole limpet hemocyanin (KLH) in chickens were earlier shown to be heritable, and to be associated with survival. Selective breeding may thus provide a strategy to improve natural disease resistance. We phenotyped 3,689 white purebred laying chickens for KLH binding NAb of different isotypes around 16 weeks of age. Heritabilities of 0.12 for the titers of total antibodies (IgT), 0.14 for IgM, 0.10 for IgA, and 0.07 for IgG were estimated. We also estimated high, positive genetic, and moderate to high, positive phenotypic correlations of IgT, IgM, IgA, and IgG, suggesting that selective breeding for NAb can be done on all antibody isotypes simultaneously. In addition, a relatively substantial non-genetic maternal environmental effect of 0.06 was detected for IgM, which may reflect a transgenerational effect. This suggests that not only the genes of the mother, but also the maternal environment affects the immune system of the offspring. Breaking strength and early eggshell whiteness of the mother’s eggs were predictive for IgM levels in the offspring, and partly explained the observed maternal environmental effects. The present results confirm that NAb are heritable, however maternal effects should be taken into account.

## Introduction

Natural antibodies (NAb) are defined as antigen binding antibodies present in individuals in the absence of immunization, vaccination, or previous infection with this antigen [1]. NAb may serve as first line of defense, likely contributing to disease resistance [2]. NAb isotypes found are IgM, IgA, and IgG [3,4]. Two NAb types are distinguished: 1) cryptic antibodies directed to self-. and altered self (neo-)antigens, which become visible after cell damage, and 2) overt antibodies directed to non-self antigens [5]. Cryptic NAb may perform homeostatic roles like clearance of cell waste, dead or metabolic materials [6], and regulation of cytokines [7]. Overt NAb likely act as an early defense barrier, preventing infection and facilitating specific immunity [2,8,9]. NAb were previously found and described in fish [10], reptiles [11], domesticated and wild birds [12,13], and various mammals [14]. In earlier studies, high NAb levels binding the overt antigen keyhole limpet hemocyanin (KLH) were related to lower mortality of layers [15,16]. Levels of NAb binding rabbit red blood cells (RRBC) [17], sheep red blood cells (SRBC) [18], and KLH [19,20] were shown to be heritable in poultry. Genomic regions underlying NAb levels were also identified before [20,21].

Modern poultry production is facing high impact changes in production systems and management. Battery cages are banned and substituted by free roaming systems, which may enhance the risk of infections. In addition, preventive use of antibiotics is strongly inadvisable, because of increasing risks for resistance in animal and human diseases. This stresses the importance of alternative ways to maintain or enhance disease resistance in poultry. Genetic selection for a higher general disease resistance might be such a strategy. For this purpose, traits reflecting disease resistance are required. These traits should be heritable, easy to measure, and related to general disease resistance. NAb titers might be a good candidate trait, but little is known of the genetic background of NAb to evaluate the opportunity of selection.

The present study describes genetic parameters of NAb binding KLH in a purebred white leghorn line population of approximately 16 weeks of age, which contained 3,689 chickens with observations for total KLH binding immunoglobulin (IgT), and the isotypes IgM, IgA, and IgG. Heritabilities, maternal effects, and genetic and phenotypic correlations were estimated within this chicken line.

## Materials and Methods

### Ethics statement

Samples and data were collected according to Institut de Sélection d’Animale (ISA) protocols, under the supervision of ISA employees. Samples and data were collected as part of routine animal data collection in a commercial breeding program for layer chickens in The Netherlands. Samples and data were collected on a breeding nucleus of ISA for breeding purposes only, and is a non-experimental, agricultural practice, regulated by the Act Animals, and the Royal Decree on Procedures. The Dutch Experiments on Animals Act does not apply to non-experimental, agricultural practices. An ethical review by the Statement Animal Experiment Committee was therefore not required. No extra animal discomfort was caused for sample collection for the purpose of this study.

### Study population

The study population was previously described by van der Klein et al. [22].

The purebred white leghorn chicken line (in other work referred to as “WA”) is a layer chicken line selected mainly for egg production. In addition, egg characteristics are included in the breeding goal.

Plasma of the studied chicken population (n = 3,689) was collected at 15 weeks of age (for males), or 19 weeks of age (for females), without anesthesia/analgesia, and was stored at -20°C until use. No chickens were killed for sample collection.

The studied chicken population originated from 314 dams. Chickens hatched at three subsequent moments with a 2 week interval (dam age: 50 to 60 weeks). Males were group housed with 12 to 14 males until wk 18 of age, and females were grouped house with 15 to 20 females until wk 18 of age. Subsequently all birds were individually housed. Chickens received a standard rearing diet 1 until wk 8, a standard rearing diet 2 from wk 8 until wk 16, and a standard laying diet from wk 16 until the end of the laying period (all commercially available diets). Feed was provided ad libitum, and water was provided ad libitum (wk 0 to 16), or 15.5 h per day (wk 16 until the end of record period). The light regime was 24 h light per day at hatch, and was weekly, gradually reduced to 19 h light per day at 11 weeks of age.

The chickens received obligatory vaccinations against Marek’s disease (d 1 intramuscular (i.m.)), infectious bronchitis (d 1, d 12–14, wk 10, wk 12 via spray; wk 16 i.m.), Newcastle disease (d 13, d 42, wk 12 via spray; wk 16 i.m.), infectious bursal disease (d 25 via spray; wk 16 i.m.), chicken anemia virus (wk 16 via water), fowl pox (wk 16 by wing web injection), and avian encephalomyelitis (wk 16 by wing web injection).

In addition, several production traits of all dams of the study population were recorded. Body weight of the dams was recorded once between 35 and 40 weeks of age. Egg production was recorded as the summed number of eggs laid between 17 and 24 weeks of age, 25 and 34 weeks of age, or 35 and 56 weeks of age. Egg weight was based on the average weight of the eggs laid on 7 subsequent days between 17 to 34 weeks of age, or 35 to 56 weeks of age. Eggs less than 30 g, and eggs over 90 g were excluded, as well as double-yolk eggs. Egg breaking strength was recorded as the average breaking strength of all eggs (maximum 6 eggs) laid during a 6 day-period between 25 to 28 weeks of age, or 35 to 56 weeks of age. Egg breaking strength was recorded within one week after collection of the last egg. Egg breaking strength was recorded using the Eggshell tester (FUTURA, Lohne, Germany) according to manufacturer’s manual. Early eggshell whiteness was based on the average of the three first eggs laid. Early eggshell whiteness was recorded with a Konica Minolta spectrophotometer, which was recorded as color values on the L*a*b* color space measurement scale. The L* value represents a black (0) to white (100) scale that was used for early eggshell whiteness measurements. Haugh unit was based on the average of three consecutive eggs laid between 17 to 34 weeks of age, or 35 to 56 weeks of age. Eggs were stored for no more than two days. Haugh unit was recorded using the Albumen height gauge (FUTURA, Lohne, Germany) according to manufacturer’s manual. Feed conversion ratio (FCR) was recorded between 33 and 43 weeks of age, and calculated as the feed intake (g) divided by the egg mass produced (g) during a measuring period of three consecutive weeks.

### Natural antibodies binding KLH

Titers of total immunoglobulins binding KLH (IgT), and the immunoglobulin isotypes IgM, IgA, and IgG binding KLH were determined in individual plasma samples by an indirect two-step ELISA as described by van der Klein et al. [22]. Briefly, flat-bottomed, 96-well medium binding plates (Greiner Bio-One, Alphen a/d Rijn, The Netherlands) were coated with 2 μg/mL KLH (Sigma-Aldrich, St. Louis, MO, USA) in 100 μL coating buffer (5.3 g/L Na_2_CO_3_, and 4.2 g/L NaHCO_3_; pH 9.6), and incubated at 4°C overnight (o/n). After washing with tap water containing 0.05% Tween 20 for 6 s, plates were tapped dry. Plasma samples were 1:10 pre-diluted (for IgT, IgM, and IgG analyses), or were 1:5 pre-diluted (for IgA analysis) with dilution buffer (phosphate buffered saline [PBS; 10.26 g/L Na_2_HPO_4_·H_2_O, 2.36 g/L KH_2_PO_4_, and 4.50 g/L NaCl; pH 7.2] containing 0.5% normal horse serum, and 0.05% Tween 20). Pre-dilutions were stored at 4°C until use the next day, or were frozen until use. Pre-dilutions were diluted with dilution buffer. Tested plasma dilution were 1:40, 1:160, 1:640, and 1:2,560 for IgT, IgM, and IgG, or 1:10, 1:20, 1:40, and 1:80 for IgA. Duplicate standard positive plasma samples (a pool of approximately half of the males) were stepwise diluted with dilution buffer. The plates were incubated for 1.5 h at 23°C. After washing, plates were incubated with 1:20,000-diluted rabbit-anti-chicken IgG heavy and light chain (IgT) labeled with horse radish peroxidase (Cat# A30-107P, RRID:AB_67386), or 1:20,000-diluted goat-anti-chicken IgM labeled with horse radish peroxidase (Cat# A30-102P, RRID:AB_66857), or 1:7,500-diluted goat-anti-chicken IgA labeled with horse radish peroxidase (Bethyl Laboratories Cat# A30-103P, RRID:AB_66833), or 1:40,000-diluted goat-anti-chicken IgG(Fc) labeled with horse radish peroxidase (Cat# A30-104P, RRID:AB_66843) (all polyclonal antibodies from Bethyl Laboratories, Montgomery, TX, USA; see also www.antibodyregistry.org), and incubated for 1.5 h at 23°C. After washing, binding of the antibodies to KLH was visualized by adding 100 μL substrate buffer (containing reverse osmosis purified water, 10% tetramethylbenzidine buffer [15.0 g/L sodium acetate, and 1.43 g/L ureumperoxide; pH 5.5], and 1% tetramethylbenzidine [8 g/L TMB in DMSO]). After 15 min (for IgT, IgM, and IgG), or 30 min (for IgA), the reaction was stopped with 50 μL of 1.25 M H_2_SO_4_. Extinctions were measured with a Multiskan Go (Thermo scientific, Breda, The Netherlands) at 450 nm. Antibody titers were calculated based on log2 values of the dilutions that gave extinction closest to 50% of E_MAX_, where E_MAX_ represents the mean of the highest extinction of the standard positive plasma samples, thereby partly correcting for plate differences [22].

The repeatability of the ELISA test was examined by randomly choosing 400 individuals of the sampled population. 1:10 pre-dilutions were made twice on two separate occasions. IgT was measured as described above in both sets of samples simultaneously using the same reagents. The Pearson correlation between the two sets was calculated using SAS 9.3.

### Statistical analyses

The following linear animal model was used for estimating variance components for IgT, IgM, IgA, and IgG titers binding KLH:

$$y_{ij}=\mu +\;P_{i}+a_{j}+e_{ij}$$ (1)

where *y*_*ij*_ is the IgT, IgM, IgA, or IgG titer, *μ* is the overall mean, *P*_*i*_ is the fixed effect of plate on which a sample was analyzed (i = 1–188 for IgT, IgM, and IgG, or i = 1–198 for IgA), *a*_*j*_ is the random additive genetic effect of the j^th^ animal assumed to be ~N(0, **A** $\sigma_{a}^{2}$), and *e*_*ij*_ is the residual term assumed to be ~N(0, **I** $\sigma_{e}^{2}$). Assumed (co)variance structures of the random model terms are **A** $\sigma_{a}^{2}$, and **I** $\sigma_{e}^{2}$, in which **A** is the additive genetic relationship matrix, $\sigma_{a}^{2}$ is the additive genetic variance, **I** is an identity matrix, and $\sigma_{e}^{2}$ is the residual variance. The pedigree used to construct **A** consisted of 4,586 individuals, and was based on 7 generations of ancestors. Note that the plate effect corrects also for other (confounded) effects on the samples, such as sex, storage, and analyses effects.

Heritabilities were calculated as

$${\text{h}}^{\text{2}}=\;\frac{\sigma_{a}^{2}}{\sigma_{p}^{2}}$$

and phenotypic variance was $\sigma_{p}^{2}=\;\sigma_{a}^{2}\;+\;\sigma_{e}^{2}$, where $\sigma_{a}^{2}$ is the additive genetic variance, and $\sigma_{e}^{2}$ is the residual variance. The likelihood ratio test was used to test whether estimated heritabilities were significant different from 0, comparing univariate model {1} to an univariate model in which the additive genetic variance was fixed at a small value of 0.0001. The likelihood ratio test was -2ln(Λ[x]), with

$$\Lambda \text{[x] = }\frac{{\text{max [L}}_{0}|\text{x]}}{{\text{max [L}}_{\text{1}}|\text{x]}}$$

where L_0_ is the likelihood under the null hypothesis with the additive genetic variance fixed at 0.0001, L_1_ is the likelihood under the alternative hypothesis without variance components constrained, and x is the given data set. Significance was assessed assuming that the likelihood ratio follows a $\chi_{1}^{2}$ distribution.

We compared univariate model {1} without a maternal effect, with an alternative model including a maternal effect {2}:

$$y_{ijk}=\mu +\;P_{i}+a_{j}+d_{k}+e_{ijk}$$ (2)

with *d*_*k*_, a random effect of the k^th^ dam, where effects of *d*_*k*_ are assumed to be ~N(0, **I** $\sigma_{m}^{2}$) with **I** as an identity matrix, and $\sigma_{m}^{2}$ as the maternal variance. The likelihood ratio test was used to test for significance of maternal environmental effects, comparing univariate model {1} without maternal effects with univariate model with maternal effects {2}. When maternal effects were significant, the heritability, and the contribution of the maternal effect was calculated as

$${\text{h}}^{\text{2}}=\;\frac{\sigma_{a}^{2}}{\sigma_{p}^{2}}\text{ and }{\text{m}}^{\text{2}}=\;\frac{\sigma_{m}^{2}}{\sigma_{p}^{2}}$$

and phenotypic variance was $\sigma_{p}^{2}=\;\sigma_{a}^{2}\;+\;\sigma_{m}^{2}+\;\sigma_{e}^{2}$, where $\sigma_{a}^{2}$ is the additive genetic variance, $\sigma_{m}^{2}$ is the maternal variance, and $\sigma_{e}^{2}$ is the residual variance. When maternal effects were significant, the likelihood ratio test was used to test whether the newly estimated heritabilities were significantly different from 0, comparing univariate model {2} to an univariate model in which the additive genetic variance was fixed at a small value of 0.0001.

Phenotypic and genetic correlations between NAb titers were estimated based on bivariate analyses using model {1}, or model {2} in case of a significant maternal effect.

To verify whether the measured traits differ between males and females, titers of males and females were treated as different traits. The likelihood ratio test was used to test whether genetic correlations were different from 1. This was done by comparing bivariate models in which all (co)variances were not fixed (except the covariance between plate in both models, which is 0) with the same bivariate model in which all (co)variances were fixed to the previously estimated (co)variances, and the covariance between the additive genetic effects was fixed so that the genetic correlation equals 1. Also heritabilities and maternal effects per sex were estimated with the previously described univariate model {1}, or model {2} in case of a significant maternal effect.

To get insight in the biological grounds of maternal effects on the offspring, production traits of the dams were added separately as fixed effects to model {2}, when maternal effects were significant. Fixed effects were tested for significance by an incremental Wald F statistics analysis. The maximum explained titer difference of significant dam production traits on NAb titers were estimated by

$$titer\;difference_{max}=\;\left(trait_{max}-trait_{min}\right)*R$$

where *trait*_*max*_ is the highest observation of a dam’s production trait and *trait*_*min*_ is the lowest observation of a dam’s production trait, and R is the regression coefficient between a dam’s production trait, and the titer of the tested NAb type of the offspring.

All statistical analyses were performed using ASReml 4.0 [23].

## Results

Descriptive statistics and heritabilities are shown in Table 1. In total 3,689 chickens, of which 1,297 males, and 2,392 females, were phenotyped for total NAb immunoglobulin (IgT) titers binding KLH around 16 weeks, and isotypes IgM, and IgG titers. Of this population 3,547 chickens, of which 1,258 males, and 2,289 females, were phenotyped for IgA, because some plasma quantities were too low. Mean titers were 7.3 for IgT, 7.5 for IgM, 6.5 for IgA, and 6.3 for IgG titers. Titers (5^th^ percentile to 95^th^ percentile) ranged from 4.9 to 9.6 for IgT, 5.4 to 9.7 for IgM, 4.4 to 8.7 for IgA, and 3.7 to 9.0 for IgG (Table 1). Heritabilities of IgT, IgA, and IgG NAb were estimated with model {1}. For IgM, significant maternal effects were detected, and therefore the heritability of IgM NAb was estimated using model {2}. The heritabilities of NAb binding KLH were estimated to be 0.12 for IgT, 0.14 for IgM, 0.10 for IgA, and 0.07 for IgG. The maternal environmental effects were estimated to be 0.02 for IgT (p = 0.08), 0.06 for IgM (p = 0.002), 0.01 for IgA (p = 0.48), and 0.01 for IgG (p = 0.30). Maternal genetic effects were not found to be significant (data not shown). Paternal (environmental and genetic) effects, which could indicate possible transgenerational epigenetic effects, were also not found to be significant (data not shown). The repeatability of the ELISA test was 0.88.

**Table 1:** Descriptive statistics, and genetic parameters (phenotypic variance $\sigma_{\text{P}}^{2}$, heritability h^2^, and maternal environmental effect m^2^) of total KLH binding natural antibody (IgT) titers, and KLH binding IgM, IgA, and IgG isotype titers in a WA leghorn chicken line at 16 weeks of age.

	IgT^a^	IgM^a^	IgA^b^	IgG^a^
Mean (SD)	7.3 (1.4)	7.5 (1.3)	6.5 (1.3)	6.3 (1.6)
Range^c^	4.9–9.6	5.4–9.7	4.4–8.7	3.7–9.0
$\sigma_{\text{P}}^{2}$(SE)^d^	1.86 (0.05)	1.27 (0.04)	1.50 (0.04)	1.68 (0.04)
h^2^ (SE)	0.12 (0.03)	0.14 (0.05)	0.10 (0.02)	0.07 (0.02)
m^2^ (SE)	NS	0.06 (0.02)^e^	NS	NS

^a^ n = 3,689, of which 1,297 males, and 2,392 females

^b^ n = 3,547, of which 1,258 males, and 2,289 females

^c^ Range shows 5^th^ quintile and 95^th^ quintile, respectively.

^d^ $\sigma_{\text{P}}^{2}$ is the phenotypic variance after adjusting for systematic environmental factors accounted for in the model.

^e^ Significant maternal environmental effect (p = 0.002)

Average NAb titers for males and females were respectively 7.1 and 7.3 for IgT, 6.8 and 7.9 for IgM, 6.4 and 6.6 for IgA, and 5.2 and 6.9 for IgG (Table 2). Although a sex effect is likely present, it was not separately taken into model {1} and {2}, because the sex effect is confounded with the plate effect. However when reanalyzing IgT in the randomly chosen repeatability set, a significant fixed effect of sex, but no significant fixed effect of plate were found. This suggests that male and female significantly differ in their IgT titers, and that analysis plate effects, other analysis effects, storage effects, or batch effects are not so much of influence on the measured IgT titers. When estimating the genetic correlation between males and females, a genetic correlation of 0.98 for IgT was found, which was not significantly different from 1. Also for IgM (0.92), IgA (0.72), and for IgG (0.85) the genetic correlation between the sexes was not significantly different from 1, but IgA almost significantly differed between sex (p = 0.06). Heritabilities between sex were (male vs. female): 0.14 vs. 0.09 for IgT, 0.17 vs. 0.15 for IgM (accounting for significant maternal environmental effects in females only), 0.15 vs. 0.11 for IgA, and 0.11 vs. 0.05 for IgG (Table 2).

**Table 2:** Descriptive statistics, and genetic parameters (phenotypic variance $\sigma_{\text{P}}^{2}$, heritability h^2^, and maternal environmental effect m^2^) of total KLH binding natural antibody (IgT) titers, and KLH binding IgM, IgA, and IgG isotype titers in males and females of a WA leghorn chicken line at 16 weeks of age.

	Males				Females			
	IgT	IgM	IgA	IgG	IgT	IgM	IgA	IgG
	(n = 1,297)	(n = 1,297)	(n = 1,258)	(n = 1,297)	(n = 2,392)	(n = 2,392)	(n = 2,289)	(n = 2,392)
Mean (SD)	7.1 (1.5)	6.8 (1.4)	6.4 (1.4)	5.2 (1.3)	7.3 (1.3)	7.9 (1.1)	6.6 (1.3)	6.9 (1.4)
Range^a^	4.7–9.6	4.7–9.2	4.1–8.8	3.2–7.4	5.4–9.5	6.1–9.7	4.6–8.6	5.0–9.3
$\sigma_{\text{P}}^{2}$(SE)^b^	2.22 (0.09)	1.75 (0.07)	1.71 (0.07)	1.71 (0.07)	1.66 (0.05)	1.00 (0.03)	1.38 (0.04)	1.66 (0.05)
h^2^ (SE)	0.14 (0.05)	0.17 (0.05)	0.15 (0.05)	0.11 (0.05)	0.09 (0.03)	0.15 (0.06)	0.11 (0.03)	0.05 (0.02)
m^2^ (SE)	NS	NS	NS	NS	NS	0.06 (0.02)^c^	NS	NS

^a^ Range shows 5^th^ quintile and 95^th^ quintile, respectively.

^b^ $\sigma_{\text{P}}^{2}$ is the phenotypic variance after adjusting for systematic environmental factors accounted for in the model.

^c^ Significant maternal environmental effect (p = 0.009)

Descriptive statistics of dam production traits are shown in Table 3. Only dam early eggshell whiteness (p = 0.004), and dam egg breaking strength (at 35–56 weeks of age) (p = 0.04) were found to significantly influence IgM. Dam early eggshell whiteness was found to explain at maximum 0.42 titer point of IgM, and dam egg breaking strength was found to explain at maximum -0.40 titer point of IgM. However, when the significance threshold value (α = 0.05) is corrected for multiple testing, only early eggshell whiteness remains significant. All other production traits were not found to be significantly associated with IgM titers. IgT, IgA, and IgG titers were not tested, because no significant maternal effect was found (see before).

**Table 3:** Number of dams (n), mean (with SD), and minimum and maximum observation (Min—Max) of the production traits of the dams of the studied population of WA leghorn chickens.

Trait	n	Mean (SD)	Min—Max	p value	R
Body weight (kg)^a^					
wk 35–40	313	1.61 (0.111)	1.28–2.01	0.79	
Egg production (#)^b^					
wk 17–24	314	19 (8.8)	0–40	0.49	
wk 25–34	314	70 (1.5)	56–71	0.80	
wk 35–56	313	152 (2.4)	133–155	0.96	
Egg weight (g)^c^					
wk 17–34	313	53.7 (2.52)	47.3–60.8	0.80	
wk 35–56	313	58.7 (2.82)	52.4–69.7	0.54	
Egg breaking strength (kg)^d^					
wk 25–28	314	4.7 (4.05)	3.7–5.7	0.84	
wk 35–56	262	4.3 (0.44)	3.0–5.7	0.04	-0.1543
Early eggshell whiteness ^e^	306	88 (1.7)	83–92	0.004	0.0471
Haugh unit^f^					
wk 17–34	264	866.9 (44.04)	739.0–996.0	0.15	
wk 35–56	313	829.1 (44.38)	636.0–962.0	0.60	
Feed conversion ratio^g^					
wk 33–43	260	1.88 (0.148)	1.54–2.38	0.83	

The p value indicates significance of the production trait when added as a fixed effects in the model of KLH binding IgM natural antibody titers at 16 weeks of age (not corrected for multiple testing). In case of significance, the estimated regression coefficient (R) is given.

^a^ Body weight of the dams was recorded once in the indicated period.

^b^ Egg production was recorded as the summed number of eggs laid in the indicated period.

^c^ Egg weight was based on the average weight of the eggs laid on 7 subsequent days in the indicated period. Eggs less than 30 g, and eggs over 90 g were excluded, as well as double-yolk eggs.

^d^ Egg breaking strength was recorded as the average breaking strength of all eggs laid during a 6 day-period in the indicated period. Egg breaking strength was recorded within one week after collection of the last egg.

^e^ Early eggshell whiteness was based on the average of the three first eggs laid. Early eggshell whiteness was recorded as color values on the L*a*b* color space measurement scale. The L* value represents a black (0) to white (100) scale that was used for early eggshell whiteness measurements.

^f^ Haugh unit was based on the average of three consecutive eggs laid in the indicated period. Eggs were stored for no more than two days.

^g^ Feed conversion ratio was calculated as the feed intake (g) divided by the egg mass produced (g) during a measuring period of three consecutive weeks.

Genetic and phenotypic correlations are shown in Table 4. Note that when estimating genetic and phenotypic correlations, maternal environmental effects were accounted for IgM. The IgT NAb titer reflects (but is not the sum of) a combination of IgM, IgA, and IgG. This is also observed in very strong genetic (0.97), and moderate phenotypic correlations (0.55) between IgT and IgM, very strong genetic (0.92), and weak phenotypic correlations (0.30) between IgT and IgA, and very strong genetic (0.96), and phenotypic correlations (0.81) between IgT and IgG. Although the genetic correlations between isotypes were very strong (IgM and IgA: 0.81, IgM and IgG: 0.86, IgA and IgG: 0.87), the phenotypic correlations were weak, but positive (IgM and IgA: 0.33, IgM and IgG: 0.26, IgA and IgG: 0.22).

**Table 4:** Estimated genetic correlations (below the diagonal) and phenotypic correlations (above the diagonal) of total KLH binding natural antibody (IgT) titers, and KLH binding IgM, IgA, and IgG isotype titers in a WA leghorn chicken line at 16 weeks of age.

	IgT^a^	IgM^a^	IgA^b^	IgG^a^
IgT	-	0.55 (0.01)	0.30 (0.02)	0.81 (0.01)
IgM	0.97 (0.03)	-	0.33 (0.02)	0.26 (0.02)
IgA	0.92 (0.07)	0.81 (0.09)	-	0.22 (0.02)
IgG	0.96 (0.03)	0.86 (0.09)	0.87 (0.10)	-

SE are shown in parentheses.

^a^ n = 3,689, of which 1,297 males, and 2,392 females

^b^ n = 3,547, of which 1,258 males, and 2,289 females

## Discussion

Selection for natural antibodies in chicken may be a promising strategy to enhance general disease resistance. NAb are involved in preventing infection [2,9], and are associated with decreased mortality in layers [15,16]. NAb were related with, and also facilitated specific antibody response levels in poultry [8,24]. NAb were previously found to be heritable in laying hens [17–20], and dairy cows [25–27] (S1 Table). SNP association studies found associations of KLH binding NAb with various immune related genes, such as interleukins, MHC, and various chemokine(-receptors) [19,21]. This suggests an immune regulating role of NAb, and a relation with regulatory networks influencing regulatory T cells, and immune balance.

In the present study, the estimated heritability for KLH binding total NAb immunoglobulin (IgT) titers was low, but significantly different from 0. Wijga et al. [17] reported a heritability for RRBC binding IgT NAb of 0.23. In addition, they reported a relative high phenotypic variance compared to this study. Difference in heritabilities in the current study and the heritability reported by Wijga et al. [17] might rest on various reasons. First, there are differences between the used populations: a White Leghorn population (present study) versus a Rhode Island Red population [17]. White Leghorns have in general less genetic diversity than Rhode Island Reds [28]. Second, the reported NAb traits differ: titers of NAb binding RRBC at 32 days of age were determined by agglutination [17], which is likely different from KLH binding NAb titers around 16 weeks of age determined by ELISA. Albeit both tests may give comparable results [29], the age of the bird does influence NAb titers [13,15,16], and thus possibly the genetic parameters. Third, the chicken population examined by Wijga et al. [17] was selected for more than 20 generations for sheep red blood cells (SRBC) specific antibodies, which are positively correlated (0.15) to RRBC NAb. Although epitope binding of SRBC is likely not similar to epitope binding of RRBC [17]. Sun et al. [19], and Sun et al. [20] estimated higher IgM, and IgG KLH binding NAb heritabilities (0.26–0.41 and 0.21–0.31, respectively) than estimated in the present study (0.14, and 0.07, respectively). As was indicated by Sun et al. [19], their data did not allow accounting for maternal effects, because of too few offspring per dam [19,20]. However, estimates of heritabilities based on maternal family relations (dam model) were higher than estimates based on an animal model, indicating possible maternal effects [19]. When in the present study maternal environmental effects were not taken into account for IgM, the heritability was 0.29, which is comparable to the IgM heritability found by Sun et al. [19]. Therefore the heritability reported by Sun et al. [19] is likely overestimated. In the present study, the heritability of IgG binding KLH was not influenced by maternal environmental effects, and is relatively low compared to Sun et al. [19], and Sun et al. [20]. The genetic different chicken populations studied (crossbred or combination of several leghorn lines), the hen-only-populations, the different sampling moments (20 or 24 weeks of age), and the maternal (environmental) effects [19,20] may account for the observed differences in heritability between the earlier studies and the present study.

As far as we know, this is the first study that estimates systemic IgA NAb heritability. The exact function of systemic IgA is unknown, although recent work suggests that systemic IgA plays a role in clearance of invading (intestinal) bacteria [30]. It can be hypothesized that systemic IgA may reflect mucosal health/immunity, e.g. mucosal IgA, since chicken secrete a substantial part of the mucosal IgA via the blood to the liver, and subsequently to the bile fluid [31,32]. However, it was also demonstrated that serum IgA has different immunological properties (e.g. pro-inflammatory) compared to mucosal IgA (e.g. anti-inflammatory) in human [33]. As far as we know, no literature elucidates the relation between systemic and mucosal IgA.

As mentioned, IgT is a combination of IgM, IgA, and IgG, which is also reflected in the estimated correlations. However, the relative contribution of IgM, IgA, and IgG to IgT is difficult to quantify. Naive B cells produce IgM, while KLH binding IgA, or IgG B cells might be the result of environmentally induced, non-antigen specific isotype switching of KLH binding IgM B cells, possibly induced via Toll-like receptors. IgM may therefore be more influenced by genetics, while IgA, and IgG may reflect immunomodulating environmental influences (f.e. vaccination), because immunization was shown to increase IgG NAb levels [34], and likely increases IgA NAB levels as well. This is also suggested by the high genetic correlation (suggesting same genetic base level), and the lower phenotypic correlation (suggesting different environmental influences or different responsiveness to environmental influences) between IgM and IgA, and IgM and IgG. This was observed as well in the significant sex effect in the repeatability test, suggesting that phenotypic NAb titers are different between males and females, possibly because of the different number of vaccinations. But the genetic correlations for IgT, IgM, IgA, and IgG between males and females did not significantly differ from 1. This means that these traits are genetically equal between sexes, even though a phenotypically difference between sexes might be present. Nevertheless the strength of the phenotypic correlations with IgT titers suggests that IgT is mainly influenced by IgG (0.81), and to a lesser extent by IgM (0.55), and IgA (0.30).

Compared to the estimated heritability, a relatively high maternal environmental effect on IgM NAb titers was found (6% of the phenotypic variance). No significant maternal effects were found on IgT, IgA, or IgG NAb titers, although reported previously [17]. This transgenerational effect on IgM titers in offspring may rest on the transfer of maternal antibodies via the egg [35]. IgM, and IgA antibodies are mainly present in the albumen of the egg, while IgG antibodies are mainly present in the egg yolk. A maternal effect on IgA could be expected if maternal antibodies (in the albumen) are the true carriers of the effect, but this effect was absent. In addition, when examining males and females separately, only female IgM NAb titers were found to be larger, and to be significantly affected by maternal environmental effects (m^2^ = 0.06, p = 0.009) compared to males (m^2^ = 0.03, p = 0.30). This could suggest that: 1) maternal IgM antibodies are not a major contributor to the maternal environmental effects, 2) female chickens are more prone to maternal environmental effects than male chickens, and 3) IgM NAb influencing genes are located on the W chromosome [20]. However, splitting up the data compromises the power to detect significant maternal effects, since number are reduced in both populations (especially in the male population). In addition, the genetic correlations for IgM between males and females did not significantly differ from 1, meaning that the traits are the same between sexes. It seems therefore unlikely that maternal environmental effects on IgM in males are absent.

To further analyze the factors that contribute to the observed maternal environmental effects, additional data of all dams of the offspring were examined. When adding these production data separately as fixed effects to model {2}, the average eggshell whiteness of the first 3 laid eggs by the dam, and the average breaking strength of 6 consecutive eggs laid by the dam between 35 and 56 weeks of age were found to be significant. It should be noted that when a correction for multiple testing is applied, only early eggshell whiteness remains significant, but not breaking strength. Early eggshell whiteness was found to explain at maximum 0.42 titer point of IgM, meaning that dams with high IgM levels produced whiter eggshells. Breaking strength was found to explain at maximum -0.40 titer point of IgM, meaning that dams with high IgM levels produced weaker eggshells in the measured period. When both effects were analyzed in one model, only early eggshell whiteness was found to be significant (p = 0.03), and egg breaking strength tended to be significant (p = 0.11). Both effects were only found to be significant when examining the whole population, but were absent or less pronounced when examined on males or females only (data not shown). This could be due to compromised power to detect the effects, because of the reduced population sizes. These production traits were not found to phenotypically or genetically correlate to IgM within the studied population of female chicken [22]. Due to an insufficient number of dams, we were not able to calculate genetic correlations between breaking strength, and early eggshell whiteness. However, within the described population, the phenotypic correlation was estimated to be -0.10, and the genetic correlations was estimated to be -0.25 (data not shown), meaning whiter eggs have a weaker eggshell, e.g. lower breaking strength. This relation has not been reported before in literature, but correlations ranging between -0.13 and -0.21 were observed in other ISA chicken populations (J. Visscher, ISA; personal communication). A possible explanation might be found in the protection of the offspring: offspring can be protected by increasing the breaking strength of the egg, and by increasing IgM titers in the offspring (possibly via maternal antibodies) with a correlated response of early eggshell whiteness. Increased breaking strength protects the egg against physical forces on the egg, and protects against pathogenic penetration of the egg. Maternal antibodies protect the egg from infection, and also protects the offspring in early life. The correlated response between early eggshell whiteness, and IgM titers in the offspring might be the result of differences in maturation of the dams by estrogen [36], or might be the result of the time the egg spent in (certain parts of) the oviduct, e.g. shell gland. Future analyses of egg data should give more insight in the relation between early eggshell whiteness, and breaking strength, and whether maternal antibodies in the egg are correlated to these. Other production parameters of the dams, e.g. egg production, egg weight, body weight, feed conversion ratio, and Haugh unit were not significantly contributing to the maternal environmental effect on IgM.

In summary, this study shows that levels of natural antibodies (IgT, IgM, IgA, and IgG) binding KLH are heritable, and provide a strategy to selectively breed for improved natural disease resistance in poultry. However, it remains to be studied whether selection for NAb might cause an (indirect) correlated response for production traits. In chickens divergently selected for primary specific antibody responses, it was found that high responders had lower body weight, decreased egg production, decreased egg weight, and later first egg production compared to low responders [37,38]. The correlated responses of NAb titers, and production parameters in the described layer chicken population are discussed elsewhere [22]. Finally, studies are in progress to determine the genetic background (SNP association studies) of NAb levels binding KLH, and whether divergently breeding for levels of NAb levels binding KLH also enhances or decrease immune responses to other antigens.

## Conclusion

The present study confirms that levels of NAb binding KLH titers around 16 weeks of age in a purebred line are heritable. Also we show that selection for total levels of immunoglobulins (IgT) binding KLH are highly correlated to IgM, IgA, and IgG isotypes binding KLH, making it possible to influence all isotypes simultaneously in the same direction by selecting for high or low NAb. However, IgM titers were relatively strongly influenced by maternal environmental effects, which should be taken into account for future studies.

## Supporting Information

S1 Table Literature overview of heritabilities of natural antibodies. Literature overview of estimated heritabilities, and maternal effects (m^2^) of total natural antibody (IgT) titers, and IgM, IgA, and IgG isotype titers binding different antigens.
(DOCX)
