# Supplementary material for: Genetic and Non-Genetic Inheritance of Natural Antibodies Binding Keyhole Limpet Hemocyanin in a Purebred Layer Chicken Line
Source: PLoS One. 2015 Jun 26;10(6):e0131088. doi: 10.1371/journal.pone.0131088 (PMC4482680; doi:10.1371/journal.pone.0131088)
Supplement: S1 Table — Literature overview of estimated heritabilities, and maternal effects (m2) of total natural antibody (IgT) titers, and IgM, IgA, and IgG isotype titers binding different antigens. (DOCX) [file pone.0131088.s001.docx]

**Supplementary Information**

**S1 Table. Literature overview of heritabilities of natural antibodies.** Literature overview of estimated heritabilities, and maternal effects ($m^{2}$) of total natural antibody (IgT) titers, and IgM, IgA, and IgG isotype titers binding different antigens. Hyphen (-) means the parameter was not estimated in that study.

| Specie | Population | Sex | Antigen^a^ | Age | IgT | IgM | IgA | IgG | m^2 b^ | Ref. |
| --- | --- | --- | --- | --- | --- | --- | --- | --- | --- | --- |
| Chicken | Crossbred | Both | RRBC | 4 wk | 0.23 | - | - | - | 0.10^c^ | [[1](#_ENREF_1)] |
|  | Crossbred | Both | SRBC | 5 wk | 0.43 - 0.03^d^ | - | - | - | - | [[2](#_ENREF_2)] |
|  | Crossbred | Female | KLH | 24 wk | - | 0.26 | - | 0.21 | - | [[3](#_ENREF_3)] |
|  | Pure lines | Female | KLH | 20 wk | - | 0.41 | - | 0.31 | - | [[4](#_ENREF_4)] |
|  |  |  |  | 40 wk | - | 0.42 | - | 0.14 | - | [[4](#_ENREF_4)] |
|  |  |  |  | 65 wk | - | 0.44 | - | 0.26 | - | [[4](#_ENREF_4)] |
| Cow | Dutch Holstein | Female | KLH | 4.3 - 6.0 yr | - | 0.25 | - | 0.25 | - | [[5](#_ENREF_5)] |
|  | Canadian Holstein | Female | KLH | Unknown | - | 0.18 | - | 0.32 | - | [[6](#_ENREF_6)] |
|  | Dutch Holstein | Female | KLH | Unknown | 0.40 - 0.34^e^ | 0.55 - 0.48^e^ | 0.40 - 0.32^e^ | 0.45 - 0.41^e^ | - | [[7](#_ENREF_7)] |

^a^ KLH: keyhole limpet hemocyanin; RRBC: rabbit red blood cells; SRBC: sheep red blood cells

^b^ When maternal (environmental and genetic) effects were not estimated, direct heritabilities can be overestimated when maternal effects are actually present.

^c^ Maternal environmental effect

^d^ Heritabilities were estimated on sire variance or dam variance, respectively. Note: estimated heritability was read out from figure.

^e^ Heritabilities were estimated on intraherd or across-herd variance, respectively.

**Literature cited Supplementary Information**

1. Wijga S, Parmentier HK, Nieuwland MGB, Bovenhuis H. Genetic parameters for levels of natural antibodies in chicken lines divergently selected for specific antibody response. Poult Sci. 2009;88(9):1805-10. doi: 10.3382/ps.2009-00064.

2. van der Zijpp AJ, Leenstra FR. Genetic analysis of the humoral immune response of white leghorn chicks. Poult Sci. 1980;59(7):1363-9. doi: 10.3382/ps.0591363.

3. Sun Y, Ellen ED, Parmentier HK, van der Poel JJ. Genetic parameters of natural antibody isotypes and survival analysis in beak-trimmed and non-beak-trimmed crossbred laying hens. Poult Sci. 2013;92(8):2024-33. doi: 10.3382/ps.2013-03144.

4. Sun Y, Biscarini F, Bovenhuis H, Parmentier HK, van der Poel JJ. Genetic parameters and across-line SNP associations differ for natural antibody isotypes IgM and IgG in laying hens. Anim Genet. 2013;44(4):413-24. doi: 10.1111/age.12014.

5. de Klerk B, Ducro BJ, Heuven HCM, den Uyl I, van Arendonk JAM, Parmentier HK, et al. Phenotypic and genetic relationships of bovine natural antibodies binding keyhole limpet hemocyanin in plasma and milk. J Dairy Sci. 98(4):2746-52. doi: 10.3168/jds.2014-8818.

6. Thompson-Crispi KA, Miglior F, Mallard BA. Genetic parameters for natural antibodies and associations with specific antibody and mastitis in Canadian Holsteins. J Dairy Sci. 2013;96(6):3965-72. doi: 10.3168/jds.2012-5919.

7. Wijga S, Bovenhuis H, Bastiaansen JWM, van Arendonk JAM, Ploegaert TCW, Tijhaar E, et al. Genetic parameters for natural antibody isotype titers in milk of Dutch Holstein-Friesians. Anim Genet. 2013;44(5):485-92. doi: 10.1111/age.12038.
